# Fe 3d Orbital Evolution in Ferrocene Ionization: Insights from ΔSCF, EOES, and Orbital Momentum Distribution

**DOI:** 10.3390/molecules30173541

**Published:** 2025-08-29

**Authors:** Feng Wang, Vladislay Vasilyev

**Affiliations:** 1 School of Science, Computing and Emerging Technologies, Swinburne University of Technology, Melbourne, VIC 3122, Australia; 2 National Computational Infrastructure, Australian National University, Canberra, ACT 0200, Australia; vvv900@nci.org.au

**Keywords:** ferrocene ionization, ΔSCF method, Fe 3d orbital characterization, excess orbital energy spectrum (EOES), energy decomposition analysis (EDA)

## Abstract

The ionization of ferrocene (Fc) remains an active topic of interest due to its complex, ulti-electron character. Accurate prediction of its first ionization potential (IP) requires methods that go beyond single-particle approximations, as Koopmans’ theorem, Janak’s theorem, and the outer valence Green function (OVGF) approach prove inadequate. Using the ΔSCF method, the first IP of Fc was calculated to be ~6.9 ± 0.1 eV, which is in close agreement with experimental values (6.72–6.99 eV). To benchmark computational accuracy, 42 models were evaluated using the CCSD, CCSD(T), and B3LYP methods with Pople and Dunning basis sets, including Fe-specific modifications to better capture 3d electron behavior. The results underscore the importance of proper treatment of Fe 3d orbitals, with B3LYP/m6-31G(d) offering the best compromise between accuracy and computational efficiency. Notably, the singly occupied molecular orbital (SOMO) in Fc^+^ is identified as the 8a_1_’ orbital, which is dominated by its Fe 3d character. This orbital, although not the α-HOMO in Fc^+^, becomes the LUMO upon ionization. Analysis of the excess orbital energy spectrum (EOES) reveals substantial energy shifts upon ionization, particularly in Fe-centered orbitals spanning both the core and valence regions. Theoretical momentum distribution (TMD) analysis of the 8a_1_’ orbital further quantifies orbital differences before and after ionization, providing complementary insights in momentum space. Finally, energy decomposition analysis (EDA) shows that while most interaction energy components become less stabilizing upon ionization, steric and Pauli terms contribute a small stabilizing effect.

## 1. Introduction

Ferrocene (Fc, di-cyclopentadienyl iron (η^5^-FeCp_2_)) stands as a cornerstone of organometallic chemistry and an iconic organometallic molecule [1], captivating the scientific community for decades [2]. As a prototypical fluxional organometallic molecule, Fc represents a historical example of “wrong but seminal” science [3], where early misconceptions led to profound advancements. Its highly symmetric structure, combined with its unusual electronic and chemical properties compared to its analogs and derivatives, has made Fc not only an esthetically intriguing molecule but also a central figure in the development of inorganic, organic, and materials chemistry. The determination of Fc’s structure [4] and ionization potential (IP) represents a landmark achievement, with enduring significance for both fundamental and applied research.

Despite its apparent simplicity, Fc—often described as a “molecular carousel”—remains a notoriously challenging system for theoretical prediction [5,6]. The quest to determine Fc’s IP has a long history [7]. Accurate calculation of Fc’s ionization potentials [1,8] and vibrational properties has consistently tested the limits of quantum chemical methods [4,9]. Early failures of Koopmans’ theorem to accurately estimate Fc’s IP [10], and subsequent difficulties faced by even advanced ab initio methods such as CASPT2 and CCSD(T), highlight the intricate electronic nature of this molecule [9].

In 2002, Ishimura et al. reviewed computational studies of Fc and concluded that “all calculations have contradicted the experimental assignment, either qualitatively or quantitatively [11]”. A decade later, Wang and co-workers [4] using combined theoretical infrared (IR) spectroscopy and a series of synchrotron-sourced experiments, confirmed that the eclipsed (D_5h_) conformer is more stable than the staggered (D_5d_) conformer in the gas phase [12,13,14,15,16]. These studies also established that the staggered (D_5d_) conformer of Fc uniquely causes the two far-IR vibrational bands to align closely [17].

Any perturbation to the staggered Fc in the gas phase—such as changes in conformation [12,17] or symmetry [18], substitutions [13,14], or solvent effects [17]—leads to a splitting of these two vibrational bands. Only the eclipsed (D_5h_) conformer of Fc and Fc^+^ is discussed in this article, as several prior experimental [19] and theoretical studies [4,5,6,8,20,21] have established that the eclipsed conformer is the more stable or dominant conformer of Fc and Fc^+^ [22].

An important property of Fc is its ability to undergo a reversible one-electron oxidation, producing the ferrocenium cation (Fc^+^) [1]. This means that the ionization of Fc results in the formation of the ferrocenium cation (Fc^+^) [7,22]. This electrochemical behavior is common among many ferrocenyl derivatives, contributing to their usefulness toward various applications [1].

Accurately determining Fc’s first IP is critical beyond academic interest. As a standard reference molecule in electrochemistry, precise knowledge of Fc’s redox behavior underpins experimental calibration and data interpretation. Furthermore, reliable theoretical benchmarks are indispensable for interpreting high-resolution synchrotron-sourced spectroscopic data and contribute to building robust machine learning-based databases for instrument development and automated data analysis [8]. Accurate theoretical predictions enable more precise assignments of spectral features, help validate experimental measurements such as photoelectron spectroscopy (PES) [23] and Penning ionization electron spectroscopy (PIES) [24], and ultimately advance the broader fields of materials science and molecular electronics.

Achieving accurate theoretical predictions for the properties of ferrocene requires not only high-level quantum mechanical methods [1,5,6,8] but also careful attention to basis set choice [4,8], especially for transition metals such as Fe [25]. Both the electronic structure method and the basis set, as well as their interplay, significantly influence the reliability of computed properties [26]. Moreover, the accuracy of a given method–basis set combination is system- and property-dependent: what works well for one molecule or property may not transfer directly to another, which is why benchmarking calculations are essential, particularly for density functional methods (DFTs). Inadequate basis sets can lead to deviations of up to ~20 kcal·mol^−1^ (~0.87 eV) in the calculated first IP compared to experiment [5,7,9]. For example, earlier ADC(3) calculations have predicted Fc’s first IP at 7.87 eV [24], significantly overestimating the experimental values (for nearly 1 eV). However, this discrepancy cannot be attributed solely to basis set limitations, as ADC(3) itself is an approximate correlated method [24]. The present study further demonstrates that even gold-standard approaches such as CCSD(T) may fail to deliver accurate IPs when paired with basis sets that do not properly account for the diffuse character of Fe 3d orbitals [27]. Thus, achieving accurate predictions requires not only advanced methods and carefully designed basis sets which are tailored for transition metal systems, but also consideration of the specific molecular system and the property under investigation. This observation is consistent with our earlier findings on the role of basis sets in electronic structure calculations of molecular properties, as discussed in the orbital momentum distribution analysis of n-butane [26].

It is essential to account for all possible corrections to the method, such as electron correlation energies, relativistic effects, basis set incompleteness, 3s/3p semi-core correlation, zero-point energy, and thermal correction to enthalpy and geometry relaxation of both the Cp^−^ rings of the Fc molecule [28,29]. Unfortunately, the resulting binding energy of Fc remains unsatisfactory, despite the acknowledged adequacy of these methods to achieve chemical accuracy. These challenges demonstrate that multiple intertwined factors—beyond the level of theory alone—influence the accurate prediction of Fc’s ionization properties [29,30].

The first IP of Fc is a fundamental electronic property that plays a crucial role in its redox chemistry, making it highly relevant across various scientific and technological fields. As a widely used reference compound in electrochemistry, an accurate determination of Fc’s IP is essential for benchmarking theoretical methods [8] and ensuring consistency in redox potential measurements.

Furthermore, Fc and its derivatives have garnered significant interest in molecular electronics, catalysis, and organometallic chemistry, where precise knowledge of their ionization behavior is critical for designing novel functional materials [7,10,11,28,31]. Despite extensive experimental and theoretical investigations, discrepancies in reported IP values persist due to variations in measurement techniques and computational approaches [7]. Therefore, a thorough and accurate study of Fc’s IP not only resolves existing inconsistencies but also provides deeper insight into the electronic structure and reactivity of metallocenes, reinforcing its importance in fundamental and applied research.

**Figure 1:**
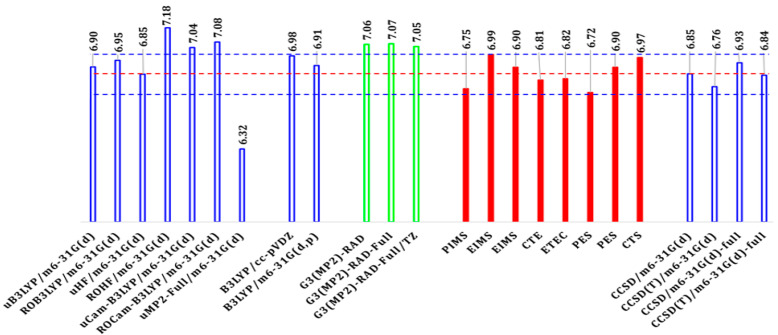
Comparison of selected calculated and measured first IPs of Fc (eV). All IP calculations in this study are carried out using the ΔSCF (or ΔDFT) method and hence are AIP. The marked horizontal red line in the figure represents the experimentally averaged IP of 6.86 eV, and the added parallel blue lines on both sides of the red line represent the experimental error bar of 6.85 ± 0.13 eV.

Recent advances in high-level quantum chemical calculations have significantly improved the determination of ferrocene’s (Fc) first IP in the gas phase. Calculations using explicitly correlated coupled cluster methods, such as CCSD(T)-F12(b) with large augmented basis sets (e.g., aug-cc-pVQZ), have yielded IP values around 6.81 eV [7], closely matching experimental measurements, which range from 6.72 eV to 6.81 eV depending on experimental conditions (see Figure 1). These approaches incorporate multiple semi-empirical correction terms to enhance basis set convergence and mitigate Hartree–Fock errors [7], playing a role analogous to modified basis sets like m6-31G* [27] tailored for transition metals such as iron (Fe).

Alternative methods, including EOM-IP-CCSD extrapolated to the complete basis set limit (CBSL) [32], have predicted vertical and adiabatic IPs (VIP and AIP) of 6.72 eV and 6.69 eV, respectively, again showing strong agreement with experimental data [7,9]. Notably, the inclusion of explicitly correlated (F12) terms and auxiliary MP2-based corrections has been critical for achieving this level of accuracy. Furthermore, fast ionization techniques such as photoelectron spectroscopy (PES) [23] and Penning ionization electron spectroscopy (PIES) [24] predominantly yield VIP values, offering additional benchmarks for theoretical validation. Building on these developments, the present study benchmarks various theoretical approaches—including DFT (B3LYP), CCSD, and CCSD(T)—in combination with multiple basis sets [4,13], with particular attention to modifications necessary for accurately treating Fe’s 3d electrons, to reliably determine the first IP of Fc.

In this study, we aim to benchmark various theoretical approaches—including DFT (B3LYP), CCSD, and CCSD(T)—in combination with multiple basis sets (including modified basis sets optimized for Fe) to determine the first IP of eclipsed ferrocene with high accuracy. Particular attention is paid to the relevance of vertical (VIP) versus adiabatic IP (AIP) in the context of fast ionization processes probed by synchrotron radiation sources. Through this benchmarking, we aim to provide theoretical support for high-precision experimental efforts and contribute valuable reference data for emerging machine-learning-based spectroscopic methods.

## 2. Computational Details

The present study performs benchmarking calculations of the first IP of Fc (eclipsed conformer, D_5h_, as it is more stable than the staggered conformer [4]). In these calculations, several post-HF ab initio methods, including Hartree–Fock (HF), second-order Møller–Plesset perturbation theory (MP2) and MP2(full), gold standard coupled cluster single and double (CCSD), CCSD(T) (valence electrons only), CCSD(T)-full (all electrons), EOM-IP-CCSD, and CCSD(T)-F12, and several DFT methods, such as B3LYP, PBE0, CAM-B3LYP, and M06-2X are employed.

For the open-shell Fc cation, ferrocenium Fc^+^, unrestricted Hartree–Fock (uHF) and DFT-based unrestricted B3LYP method (uB3LYP) methods are employed, together with the restricted open-shell B3LYP (ROB3LYP) levels of theory. In a study using complete basis sets (CBS) on intermolecular interactions of S22 set of small to medium-sized molecules consisting of first- and second-row atoms, Head-Gordon and co-workers [33] indicate that the B3LYP/CBS achieves even better binding energy than the gold standard CCSD(T)/CBS of Burns, Marshall and Sherrill [34].

It is well known that transition metals such as Fe require additional attention in basis set [8,25,27], due to their complex electronic structures and unique bonding characteristics. For example, transition metals have partially filled d-orbitals, which lead to strong electron correlation effects. Standard basis sets may not adequately capture these interactions, necessitating the use of larger, more flexible basis sets or explicitly correlated methods. As a result, specialized basis sets like cc-pVnZ (with added core correlation functions) [35], def2-TZVPP [36,37], or metal-specific basis sets such as 6-31G(d)/LanL2TZf (here Fe (transition metals) for LanL2TZf and 6-31G(d) for other atoms) [1] and m6-31G(d) (only the basis set of Fe is modified whereas other atoms are 6-31G(d) basis set) [25,27] are often required to achieve accurate computational results.

It is important to include a moderately diffuse d function for the first-row transition metals such as Fe, and m6-31G(d) has been shown to reproduce the relative energies of the most important configurations in all first-row transition metal atoms and their singly charged cations [25,27]. Research has revealed that, for transition metals such as Fe, both atomic configurations of 3d^n^4s^1^ and 3d^n−1^4s^2^ are important [38] and must be included in the basis set [25,27], in order to more appropriately describe the Fe-contained complexes such as Fc [4,39].

The original 6-31G* basis set was developed by Wachters’ 14s9p5d third-row basis set [40] augmented with an additional diffused function according to Hay [40], and with experiment. The modified 6-31G* basis set (m6-31G*) [27], introduces a moderately diffuse d-function to better describe the energetics of the 3d orbitals in open-shell and low-spin systems, including metals from Sc to Cu [25]. The present B3LYP/m6-31G(d) model is approximately equivalent to B3LYP/6-31G(d)/LanL2TZfp of Toma et al. [1].

To benchmark the suitability of the m6-31G(d) basis set in the study of Fc, in which only the Fe basis set in the 6-31G(d) is modified, several commonly used basis sets, such as Fe [20,32] modified Pople basis sets, m6-31G(d,p), m6-31++G(d,p), m6-31++G(2d,2p), m6-31++G(3d,3p), m6-31++G(3df,3pd); Dunning basis set series of cc-pVDZ, cc-pVTZ, cc-pVQZ and cc-pV5Z; and aug-cc-pVDZ, aug-cc-pVTZ, aug-cc-pVQZ and aug-cc-pV5Z; and the Fe modified Dunning basis sets of m(aug-cc-pVDZ), m(aug-cc-pVTZ), etc., are considered in the benchmarking calculations for Fc.

Due to significant computational costs, most of the basis sets are combined with the B3LYP level of theory; the Dunning basis set series is also performed at the highest possible level of theory, that is, CCSD and CCSD(T) levels. The present study employs the complete basis set (CBS) methods based on the methods of Halkier et al. [41] and Helgaker et al. [42] All first IP calculations in this study are carried out using the ΔSCF (or ΔDFT) method, as

IP_1st_ = E_tot_ (Fc^+^) − E_tot_ (Fc)

This is, in fact, the adiabatic IP (AIP) as both the geometries of Fc and Fc^+^ are optimized in the gas phase, respectively, which reduces a possible source of error due to solvent effect and solvent models. Note that VIP calculation is based on the geometry of neutral Fc for both Fc and Fc^+^ cation calculations, which is a good approximation for fast ionization processes. It was indicated that the Koopman Theorem fails to calculate ionization potentials of Fc [8,10] due to the strong relaxation effect upon ionization of a d-electron.

The orbital theoretical momentum distribution (TMD) calculations used the same scheme detailed in Liu et al. [43]. All quantum mechanical calculations were based on the optimized geometry of eclipsed Fc (and Fc^+^) using the B3LYP/m6-31G(d) model (Fc), and the uB3LYP/m6-31G(d) model for Fc^+^ were performed using the Gaussian16 computational chemistry package [44].

Finally, energy decomposition analysis (EDA) [45] using the extended transition state (ETS) method [46] is calculated using the Amsterdam density functional (ADF) computational chemistry package [47].

## 3. Results and Discussion

### 3.1. First IP of Fc—Measurements vs. Calculations

The electron configuration of the ground electronic state of Fc^+^ has been a long debate with continuing interest [24,38]. It has been established that the geometrical properties are not greatly enhanced in the ion (and in the solid) from the gas phase of Fc [36]. The Fc^+^ cation also prefers the eclipsed (D_5h_) conformer of Fc^+^. There is a subtle energy difference between the frontier occupied orbitals (4e_2_’ and 8a_1_’) of Fc [24].

Incorporating the electron correlation effect and using an appropriate basis set for the transition metal iron (Fe) in theoretical methods is very important for accurately predicting the properties of Fc [4,7,8]. There are no direct measurements for isolated Fc^+^ alone; thus, the accuracy and applicability of theoretical methods for Fc/Fc^+^ are evaluated using measurable properties of the pair [7,8]. These properties include geometric properties such as C-H, C-C, and Fe-Cp distances and bond angles of neutral Fc, rotational energy barrier, vibrational frequencies such as inferred (IR) frequencies and first IP which are given in Table S1 of the Supplementary Materials (SM). The present (U)B3LYP/m6-31G(d) calculations exhibit the valence electron configurations as:

Fc (X^1^A_1_’): (core) … (3e_2_’)^4^ (3e_2_’’)^4^ (6a_2_”)^2^ (4e_1_”)^4^ (6e_1_’)^4^ (8a_1_’)^2^ (4e_2_’)^4^ (5e_1_”)^0^

Fc^+^ (X^2^A_1_’): (core) … (3e_2_’)^4^ (3e_2_”)^4^ (6a_2_”)^2^ (8a_1_’)^1^ (4e_1_”)^4^ (6e_1_’)^4^ (4e_2_’)^4^ (5e_1_)^0^

This is consistent with the results of our earlier study [4] and Liu et al. [43] for Fc. In the Fc^+^, the electron removed is a β-electron. The results align with those commonly observed in complexes that contain transition metals [1,8]. In the case of ferrocene, the highest occupied molecular orbitals (HOMOs) are largely Fe-centered but may involve two cyclopentadienyl rings [4]. In agreement with earlier studies, the Fe d-electrons dominate the frontier orbitals, and in all neutral ferrocenes, the calculated HOMO is of 3d_xy_ character [1,4]. Upon oxidation, a β-electron is removed from the HOMO (4e_2_’) of the ferrocene molecule. Following ionization, the orbital dominated by the 3d_z_^2^ orbital of Fe becomes the singly occupied molecular orbital (SOMO) in the α-spin configuration of the resulting open-shell ferrocenium cation (Fc^+^). This orbital remains distinct in both the neutral and oxidized states and serves as the characteristic orbital associated with ferrocene ionization [8].

The reason that Koopmans’ theorem is not valid for the calculation of the IPs of ferrocene remains unresolved even after several decades. Different extent of the electronic rearrangement which occurs upon ionization depends on the nature of the orbital involved in the ionization process [1,10,11]. A growing theoretical and experimental evidence shows that in open-shell molecules the molecular orbital accommodating the unpaired electron (the singly occupied molecular orbital, or SOMO) is no longer energetically the HOMO [1,5]. Such systems can be found in quantum dots, transition metal complexes and even organic molecules.

Figure 1 compares the first IP of Fc calculated using the B3LYP, CCSD, and CCSD(T) levels of theory with several basis sets. The calculated IPs of Fc are compared with available theoretical and experimental results. Table S2 in the Supplementary Materials (SM) reports the calculated first IPs of Fc using various models and the available experimental IPs of the same compound. The basis sets in this study have been detailed in the previous section. For the detailed comparison of the basis sets, refer to Table S3 in Supplementary Materials.

From Figure 1, it is evident that the measured IP of Fc depends on the techniques and conditions employed. Specifically, it varies between 6.72 eV obtained through photoelectron spectroscopy (PES) and 6.99 eV using electron impact mass spectroscopy (EIMS) (the blue horizontal lines), and the experimental IP range is given as 6.86 ± 0.13 eV (the red horizontal line). Moreover, even using the same technique, such as PES, the IP of Fc also depends on the conditions of the experiments. For example, the IP of Fc is 6.72 eV according to Rabalais et al. but it is estimated to be 6.90 eV by Gleiter et al., with both using the PES technique. Nevertheless, all reported measurements of the first IP of Fc are not larger than 7.00 eV.

Details of the IPs in Figure 1 are given in Table S2 in the SM. This table lists the available first IPs of Fc obtained from the present study, alongside several theoretical methods and experimental measurements. A significant discrepancy of over 1 eV between experimental and calculated data was found for some methods, as noted by Toma et al. [1]. For example, the measured first IPs of Fc do not exceed 7.00 eV. In theory, high-level ab initio calculations such as ADC(3) [30] and (Δ)MP2 [8] produces the IP above 7 eV except for SAC-CI (6.26 eV) [11], whereas the ΔDFT and ΔHF models produce the IPs as 6.90 eV and 6.85 eV (for eclipsed Fc), respectively, in excellent agreement with the measurements. Hence, the first IP of Fc can be accurately calculated using the ΔSCF methods. The cancelation observed in the ΔSCF results suggests that there are certain structural similarities between Fc^+^ and Fc, indicating significant relaxation energy after ionization.

Most experimental techniques for measuring ionization energies, such as Photoelectron Spectroscopy (PES), Pinning Ionization Electron Spectroscopy (PIES), and Electron Ionization Mass Spectrometry (EIMS), operate on very short timescales. As a result, the measured ionization potential corresponds to vertical ionization (VIP), as the generated cation does not have enough time to undergo structural relaxation.

In contrast, ionization potentials calculated using Eq(1) typically represent the adiabatic ionization potential (AIP), whereas values obtained via Koopman’s theorem (when applicable) correspond to VIP. For instance, the VIP of ferrocene (Fc) is approximately 0.08 eV higher than its AIP, as reported by Makos et al.

### 3.2. Benchmark Theoretical Models for First IP of Fc

**Figure 2:**
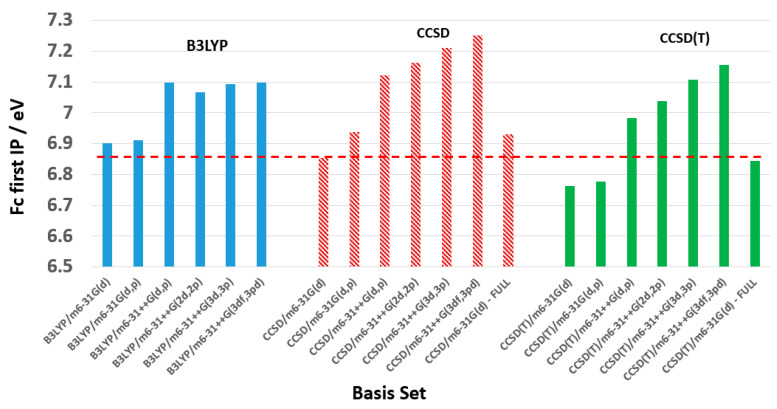
Comparison of B3LYP and the gold standard CCSD and CCSD(T) with the basis set. The averaged experimental result at IP = 6.85 eV is marked by the red dashed line.

The present study benchmarks the level of theory and basis sets for calculations of the first IP of Fc. As the m6-31G(d) basis set, when combined with the DFT B3LYP functional, accurately predicted the correct conformer from their far-IR spectra, it also shows one of the best performances in Figure 1. When combined with the gold standard CCSD, CCSD(T)-full (all electrons), this basis set achieves the optimal agreement (close to the horizontal red line), as shown in Figure 1. However, variations in the B3LYP functional, such as CAM-B3LYP, introduce larger error bars. As a result, Figure 2 compares the three levels of theory, such as B3LYP, CCSD, and CCSD(T) in combination with the modified (on Fe) basis set of Pople’s series with polarization functions and defuse functions, respectively.

The models that utilize the m6-31G(d), m6-31G(d,p), and m6-3G(d)-full basis sets show good agreement in the ionization potential (IP) for Fc. Figure 2 and Figure S2 compare the first IP of Fc produced using the CCSD and CCSD(T) levels of theory with various basis sets. As seen in Figure 2, the nine models analyzed include B3LYP/m6-31G(d), B3LYP/m6-31G(d,p), CCSD/m6-31G(d), CCSD/m6-31G(d,p), CCSD/m6-31G(d)-full, CCSD(T)/m6-31G(d), CCSD(T)/6-31G(d,p), CCSD(T)/m6-31++G(d,p) and CCSD(T)/m6-31G(d)-full. Among these, the CCSD/m6-31G(d) and CCSD(T)/m6-31G(d)-full models yield the most accurate IPs for ferrocene (6.854 eV and 6.844 eV, respectively). These values are nearly identical to the experimental value (indicated by the red dashed line). Next in accuracy are the B3LYP/m6-31G(d) (6.902 eV) and B3LYP/m6-31G(d,p) (6.912 eV) models. Notably, all the top-performing models use the m6-31G(d) basis set without any corrections to the IP of Fc.

**Figure 3:**
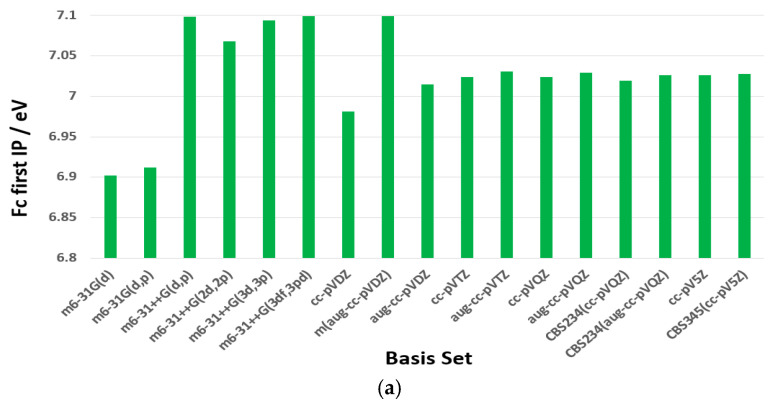

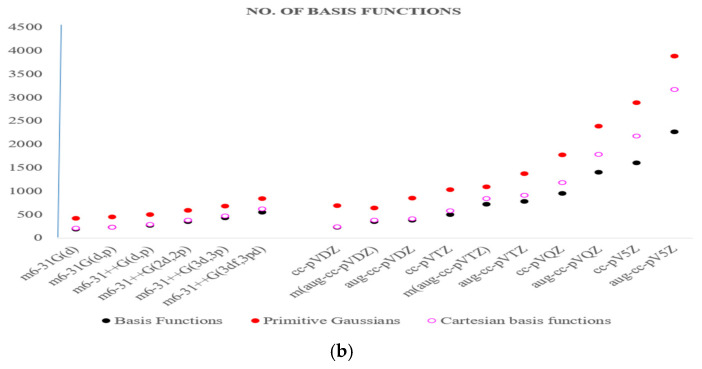
(**a**) Benchmarking of the first IP of Fc based on B3LYP with various basis sets; larger basis sets generally overestimate the IP of Fc. (**b**) Total number of basis functions (blue) and total number of primitive gaussians (red) of each basis set.

Figure 3a compares the first ionization potential (IP) of ferrocene (Fc) calculated using the B3LYP functional with various basis sets, while (b) provides an overview of the basis set sizes. As shown in the figure, larger basis sets do not always yield more accurate IP values for Fc. However, they do contain more basis functions and primitive Gaussians, leading to significantly higher computational costs. For instance, the m6-31G(d) basis set consists of 194 basis functions and 424 primitive Gaussians, and when combined with B3LYP, it predicts an IP of 6.902 eV. In comparison, the m6-31++G(3df,3pd) basis set employs 554 basis functions and 844 primitive Gaussians, yielding a slightly higher IP of 7.099 eV, but at a much greater computational cost.

Similarly, the B3LYP/cc-pVDZ model estimates the IP at 6.981 eV, which is more accurate than the values obtained using the B3LYP/cc-pVTZ (7.024 eV) and B3LYP/cc-pVQZ (7.024 eV) models. Despite this, cc-pVDZ contains only 233 basis functions, whereas cc-pVTZ and cc-pVQZ require 508 and 783 basis functions, respectively, further emphasizing the trade-off between accuracy and computational efficiency. Figure S1 in the SM provides more details of the basis set information.

**Figure 4:**
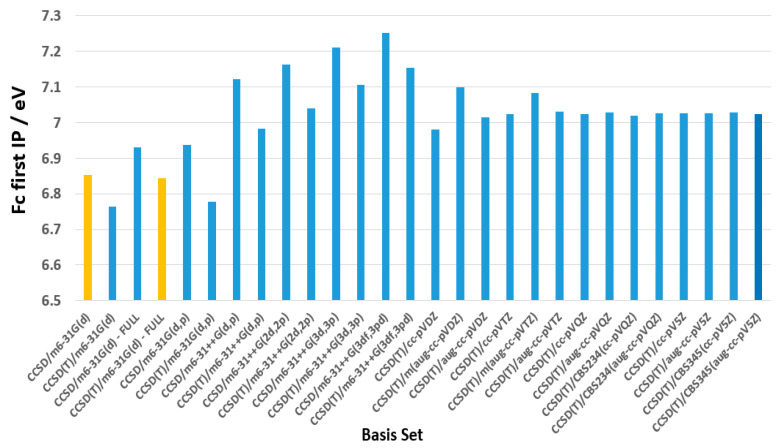
Benchmarking the calculated first AIP of Fc using various levels of theory and basis sets.

Figure 4 presents benchmark calculations of the first IP of Fc using various theoretical methods and basis sets. The performance of these models is evaluated in terms of accuracy, computational efficiency, and consistency with high-level theoretical and experimental results. Among the basis sets used, m6-31G(d) is the smallest (194 basis functions) and the smallest Dunning-type basis set is cc-pVDZ (233 basis functions) (see Table S3 in Supplementary Materials). Although Dunning’s (aug-)cc-pVXZ basis sets provide stable IP predictions, they tend to be less accurate for Fc, as these basis sets were not specifically optimized for iron (Fe). In contrast, the m6-31G family of basis sets has been explicitly optimized for Fe, starting with 6-31G and extending to 6-31G(d) (m6-31G(d)) and 6-31G(d,p) (m6-31G(d,p)). As seen in Figure 3, the B3LYP/m6-31G(d) model provides a strong balance between accuracy and computational efficiency, making it an appealing choice for studying Fc. This conclusion is further reinforced by a benchmarking study on DFT functionals for predicting [1] Fe Mössbauer spectral parameters, where Bochevarov et al. identified B3LYP and O3LYP as the best-performing functionals among more than half a dozen that were tested [48].

In Figure 4, the calculated first adiabatic IP of Fc is compared across different levels of theory (see Table S2 in Supplementary Materials for detailed values). Notably, the B3LYP/m6-31G(d) IP surpasses even the complete basis set (CBS) limit calculations, such as CBS234(cc-pVQZ) for 7.019 eV, CBS234(aug-cc-pVQZ) for 7.026 eV, CBS345(cc-pV5Z) for 7.028 eV and CBS345(aug-cc-pV5Z) for 7.025 eV. Additionally, a recent B3LYP/6-31G(d)/LanL2TZf calculation using the ΔSCF method reported an IP of 6.92 eV for Fc [8]. The CCSD(T)/m6-31G(d)-full model predicted an IP of 6.84 eV, which is in excellent agreement with the CCSD(T)-F12/aug-cc-pVQZ calculation (6.81 eV) by Zhao et al [7]. Furthermore, the EOM-IP-CCSD/CBS calculations by Makos et al. [32] yielded 6.67 eV for the vertical IP (VIP) and 6.59 eV for the adiabatic IP (AIP).

While CCSD(T) calculations are often considered the gold standard for IP predictions, their high computational cost is a major limitation. These calculations require significant memory and CPU time, making them impractical without access to high-performance computing resources. For example, a single-point energy calculation using B3LYP/m6-31G(d) takes only 22.3 s for Fc (eclipsed), whereas the corresponding uB3LYP/m6-31G(d) calculation for Fc^+^ requires 118.8 s—approximately five times longer. Similarly, a CCSD(T)/m6-31G(d) calculation takes 2710.75 s (45.18 min) for Fc, while the uCCSD(T)/m6-31G(d) calculation for Fc^+^ requires 14,772.75 s (246.21 min)—over five times longer (The CPU time mentioned here reflects the use of two Intel Xeon E5-2650 8-core CPUs at 2.00 GHz, totaling 16 cores).

Furthermore, switching from m6-31G(d) to the Dunning aug-cc-pVDZ basis set nearly doubles the computational cost. For instance, a CCSD(T)/aug-cc-pVDZ calculation for Fc takes 5160.5 s (86.01 min), compared to 2710.75 s (45.18 min) for CCSD(T)/m6-31G(d). However, this additional cost does not necessarily lead to improved accuracy—the CCSD(T)/aug-cc-pVDZ model introduces a 2.60% error in the calculated IP, whereas the error using B3LYP/m6-31G(d) is only 0.61%. The results obtained in this section for the first ionization potential (IP) calculations of ferrocene (Fc) reveal an important aspect of accurately computing this value: the treatment of the Fe 3d electrons. These electrons are particularly sensitive to the choice of basis set.

Unlike larger correlation-consistent basis sets, such as the aug-cc-pV5Z, which are optimized for post-Hartree–Fock correlated methods, our benchmarking results show that the m6-31G* basis, in which only the Fe basis is modified while the rest of the molecule retains the standard 6-31G* description, yields the first IP of Fc in excellent agreement with experiment. This accuracy is attributed to its ability to capture the subtle relaxation and polarization effects of the Fe 3d orbitals upon ionization, without over correlating core or diffuse orbitals that contribute little to the IP. Although larger correlation-consistent basis sets offer formal convergence toward the CBS limit, they do not necessarily provide better agreement with experimental results within DFT frameworks unless paired with appropriately scaled functionals. Our results thus highlight that basis set functional compatibility, rather than nominal size or completeness, is crucial for transition metal systems like Fc. In this context, the B3LYP/m6-31G(d) emerges as the most promising model for predicting properties of ferrocene and its derivatives [4].

### 3.3. Fe 3d Electron Dominance at Ionization

The ground electron configurations of Fc^+^ and neutral Fc differ, particularly in the outer valence space with orbital 8a_1_’ being HOMO-1 in Fc, which becomes the singly occupied molecular orbital (SOMO, 8a_1_’) in Fc^+^ upon ionization and HOMO-3. Although the IP theorem within the Kohn–Sham (KS) framework of DFT has known limitations, the use of KS orbital energies remains a reasonable approximation, particularly within closely related classes of compounds [1]. It is noted that KS orbital energies can be related to experimental IPs (IPₑₓₚ) through a constant shift [1], with the energy of the highest occupied KS orbital (Janak Theorem [49]) showing a linear correlation with IPₑₓₚ. As indicated by Toma et al., the calculation of the orbital energies of ferrocene and its derivatives may be a useful tool to understand the electronic structure of Fc and Fc^+^.

**Figure 5:**
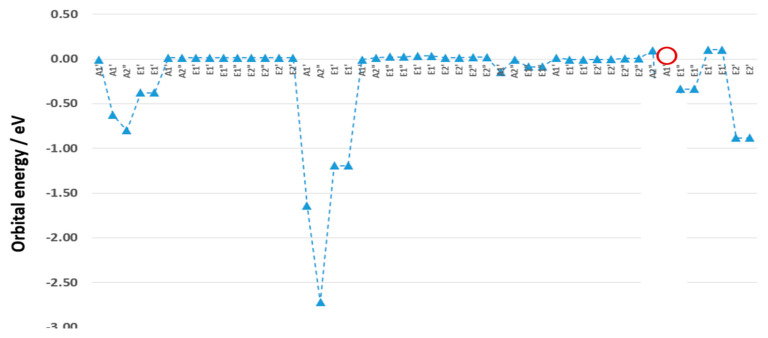
The EOES of α- and β-electrons of Fc^+^, calculated using UB3LYP/m6-31G(d) level of theory. It is assumed that a β-electron is removed from the molecule.

Figure 5 reports the excess orbital energy spectrum (EOES) [38] between α- and β-electrons of Fc^+^ of the eclipsed conformer. The ∆ε indicates how different each orbital energy of Fc^+^ is on a one-on-one electron basis (note that all the doubly degenerate orbitals are counted twice in Figure 5, and 8a_1_’ orbital becomes the HOMO-3 in Fc^+^). Upon ionization, all electrons change their energies in the ranges of |∆ε| < 3.0 eV with most energy differences between α- and β-electrons are negligible, except the Fe-dominant orbitals of Fe (2s, 2p_z_, 2p_x_, 2p_y_) for the first group of orbitals 2-5; the next group of Fe dominant orbitals are Fe (3s, 3p_z_, 3p_x_, 3p_y_) for the next group of orbitals 16–19; and the last group of orbitals are in the outermost valence orbitals, with three pairs of doubly degenerate orbitals and 8a_1_’ orbital, due to electron energy density delocalization.

The HOMO (4e2’) and the SOMO (8a_1_’) are dominated by Fe-3d electrons [43]. This is the case when the orbitals upon ionization are dominated by the orbitals of transition metals (Fe) [16,39,50].

The outermost (Group 3) orbitals of Fc^+^ will be the focus due to the variation in both the orbital order and energies, unlike Group 1 and Group 2 orbitals, which exhibit excess orbital energy changes. At ionization, the SOMO is orbital 8a_1_’, which is dominated by the Fe 3d_z_^2^ orbital. Further analysis of the SOMO (α–8a_1_’), it is found that the d-electrons (d_z_^2^) of Fe dominate the SOMO, with 97.3% to the total density of Fe in Fc, the s-electrons of Fe contribute to the balance of 2.7%, whereas the contributions from the p-electrons of Fe can be neglected in SOMO. This is also reflected in the SOMO of Fc^+^ in Figure 5. It is noted that the SOMO of Fc^+^ is not necessarily the HOMO in the α-electron configuration and the LUMO in the β-electron configuration in open-shell Fc^+^, in agreement with Toma et al. [1] using B3LYP/6-31G(d)/LanL2TZfp level of theory.

The SOMO (8a_1_’) orbital is distinctive in both neutral and cation forms and represents the signature orbital of Fc^+^ and is dominated by the Fe-3d_z_^2^ orbital [39], in agreement with experimental data [43] and previous results [1]. In a combined experimental and theoretical study of Liu et al. [43] on Fc ionization, it was suggested that the frontier orbitals of neutral Fc 4e’ (HOMO) and 8a_1_’ (HOMO-1) are indeed dominated by 3d electrons of Fe by 83.69% and 89.37%, respectively [43].

**Figure 6:**
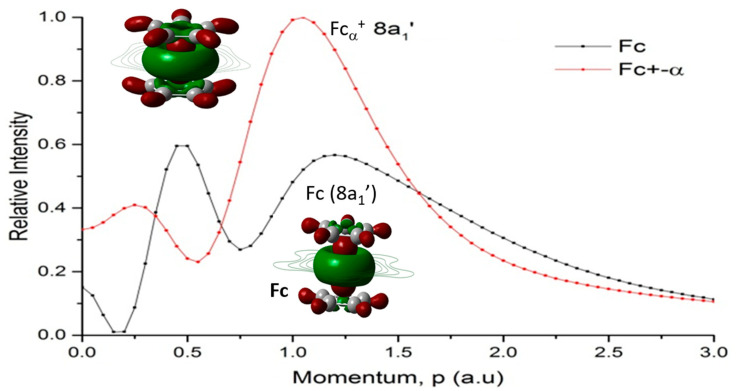
Comparison of theoretical momentum distribution (TMD) profile of orbital 8a_1_’ for the α-electron of Fc^+^ and Fc. The Dyson orbital electron charge distributions of 8a_1_’ of Fc^+^ (α-electron, red) and 8a_1_’ orbital of Fc (black).

Further analysis of this orbital is carried out using the Dyson orbital theoretical momentum distribution (TMD) [43]. Figure 6 compares the Dyson orbital TMD 8a_1_’ of Fc and Fc^+^(α-electron in the SOMO). This orbital (8a_1_’) is not the HOMO in both Fc and α-Fc^+^ but locates the hole on Fc^+^ and becomes the LUMO in β-Fc^+^.

Orbital 8a_1_’ (HOMO-1 for Fc or HOMO-3 for α-Fc^+^) is dominated by the Fe-3d_z_^2^ orbital, which aligns along the directions of the z-axis, i.e., the Cp-Fe-Cp axis. Figure 6 presents the orbital theoretical momentum profiles (TMP) for those orbitals. The TMDs of 8a_1_’ provide additional information about the orbitals in momentum space quantitatively from the information obtained from the coordinate space [26].

Although the electron density of this orbital in Fc^+^ (α) and Fc appears almost identical in coordinate space, the orbital TMD of Fc^+^ (α) and Fc show notable differences in momentum space quantitatively. The red profile (Fc^+^) and black profile (Fc) of 8a_1_’ are very interesting, which exhibit significant quantitative differences in the d-electron dominant component (Fe 3d_z_^2^) [39]. The large differences in the small momentum region of *p* < 1.5 au indicate that the orbitals (8a_1_’) are very different in their long-range region.

### 3.4. Energy Decomposition Analysis of Fc^+^

The ionization of ferrocene induces notable changes in its interaction energy components, which can be revealed through energy decomposition analysis (EDA) [45]. The interaction energy ΔE_Int_ term can be decomposed into three important quantities that have direct physical meaning [16] of electrostatic (ΔE_Estat_), Pauli (ΔE_Pauli_) and orbital (ΔE_Orb_) energies.

**Figure 7:**
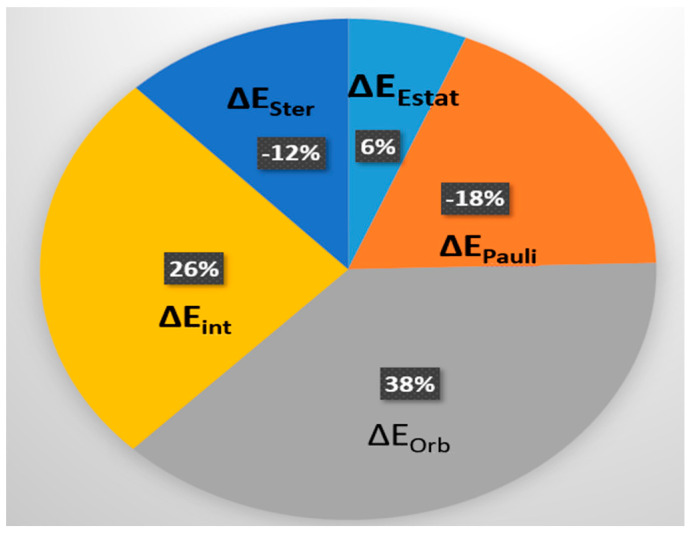
Comparison of energy components between Fc^+^ and Fc in kcal∙mol^−1^ calculated using UB3LYP/TZ2P+ and B3LYP/TZ2P level of theory and the ETS method [46]. Here the pie plot is based on ∆∆E_i_ = ∆E_i_ (Fc^+^) − ∆E_i_ (Fc), where the energies (eclipsed) are obtained from [16].

Figure 7 reports the EDA of Fc^+^ with respect to neutral Fc, using the extended transition state ETS method [46] based on the Amsterdam density functional (ADF) computational chemistry package [47]. Comparing the total interaction energies (ΔE_Int_) between Fc and Fc^+^, a consistent destabilization of approximately 26% (161.5 kcal∙mol^−1^) is observed in both conformers, reflecting the overall energetic cost of electron removal.

This destabilization is primarily driven by a reduction in the orbital interaction energy (ΔE_Orb_), which increases by 38% (237.81 kcal∙mol^−1^) in the eclipsed conformer. Such an increase indicates a significant loss of covalent bonding character, particularly involving Fe-centered orbitals, as one electron is removed from a delocalized molecular orbital during ionization.

At the same time, the Pauli repulsion (ΔE_Pauli_) decreases markedly upon ionization—by −18% (115.75 kcal∙mol^−1^)—reflecting a reduction in electron crowding within the molecular framework. The electrostatic component (ΔE_Estat_) also becomes less stabilizing, increasing by 6% (39.4 kcal∙mol^−1^). These results highlight that ionization not only weakens orbital interactions but also reduces both attractive electrostatics and repulsive exchange contributions.

Interestingly, the steric energy (ΔE_Ster_), which reflects geometric and spatial interactions, becomes more stabilizing upon ionization, decreasing by 12% (76.35 kcal·mol^−1^). This suggests that ionization facilitates a relaxation of steric strain, likely due to subtle geometric adjustments such as changes in Fe–Cp distances or reduced repulsion between Cp rings. When comparing the two conformers of Fc^+^ directly, the eclipsed form exhibits slightly larger changes in both orbital and electrostatic components, along with more favorable steric relaxation. As a result, it achieves a marginally more stabilizing interaction energy. Although the total energy difference between eclipsed and staggered Fc^+^ is small, the decomposition of individual energy components reveals a subtle shift in the factors governing conformational preference.

In neutral Fc, the preference for the eclipsed form arises from a delicate balance in which large Pauli repulsion is nearly canceled by stabilizing orbital and electrostatic interactions. In contrast, in Fc^+^, this balance shifts: electrostatics and steric effects, although weaker overall, become the dominant stabilizing forces, while orbital interactions are diminished or even slightly destabilizing. These findings underscore that ionization not only alters the electronic structure of ferrocene but also fundamentally reshapes the energetic landscape that determines its conformational behavior.

## 4. Conclusions

This study systematically assessed a broad range of post-Hartree–Fock (HF) and density functional theory (DFT) methods for predicting the first ionization potential (IP) of ferrocene (Fc), in conjunction with Pople and Dunning basis sets—both standard and modified for transition metal accuracy. The results demonstrate that accurate IP prediction requires not only a method capable of capturing electron correlation and orbital relaxation, but also a basis set refined to appropriately describe the Fe 3d orbitals. Among all approaches tested, the ΔSCF method proved most reliable, as it explicitly accounts for relaxation effects between the neutral and ionized states. The best agreement with experimental IP values (6.72–6.99 eV) was achieved using the B3LYP/m6-31G(d) model, which offered a strong balance between accuracy and computational efficiency.

Our analysis reveals that the ionization of Fc is primarily an all-electron process centered on the Fe atom. This process involves the removal of an electron from the Fe 3d-dominated 8a_1_’ orbital. Notably, this orbital, which is not the HOMO in the α-spin channel of Fc^+^, becomes the LUMO after ionization. This indicates a significant reordering of the frontier molecular orbitals.

Excess orbital energy spectrum (EOES) analysis confirms that Fe-centered α-electrons, particularly those with axial (z-direction) symmetry, undergo the largest energy shifts during ionization—consistent with Mössbauer EFG experimental data. Theoretical momentum distribution (TMD) analysis further supports this, capturing quantifiable differences in the 8a_1_’ orbital before and after ionization. Additionally, energy decomposition analysis (EDA) shows that while electrostatic and orbital components become less stabilizing in Fc^+^, a modest stabilizing contribution arises from steric and Pauli terms. This is likely due to reduced electron repulsion and subtle geometric relaxation.

In summary, our findings reinforce that ionization in Fc is fundamentally a metal-centered electronic event. It requires both orbital relaxation and accurate Fe 3d representation to model correctly—conditions uniquely fulfilled by the ΔSCF approach with appropriately tailored basis sets. These insights may also be applicable to other transition metal sandwich complexes and guide future modeling of metal-centered redox processes. We also examined other first-row transition metal metallocene complexes to investigate their property trends, and these results will be reported in a forthcoming publication.

## Data Availability

Data are contained within the article and Supplementary Materials.

## Supplementary Materials

The following supporting information can be downloaded at: https://www.mdpi.com/article/10.3390/molecules30173541/s1. See supplementary material for more results on ferrocenium including optimized geometric parameters, detailed benchmarking calculation results of first ionization potential (IP), and information about the basic set used for benchmarking calculations. Table S1: Geometric properties of Fc+ (both conformers) using different models; Table S2: Bench-marking calculations for first IP (eV) of ferrocene using B3LYP, CCSD, and CCSD(T) and various basis sets; Table S3: Summarized information of the basis sets in the benchmarking calculations; Figure S1: Summarized comparison about number of basic functions in the benchmarking calcula-tions; Figure S2: Comparison of the gold standard CCSD and CCSD(T) level of theory with various basis sets for the first AIP of Fc. The results (6.85 eV) with gold colors exhibit the best agreement with the recognised measurement.
