# Supplementary material for: Fe 3d Orbital Evolution in Ferrocene Ionization: Insights from ΔSCF, EOES, and Orbital Momentum Distribution
Source: Molecules. 2025 Aug 29;30(17):3541. doi: 10.3390/molecules30173541 (PMC12430873; doi:10.3390/molecules30173541)

Fe 3d Orbital Evolution in Ferrocene Ionization: Insights from ΔSCF, EOES, and Orbital Momentum Distribution

Feng Wang[1,*] and Vladislay Vasilyev[2]

1Department of Chemistry and Biotechnology, Swinburne University of Technology, Hawthorn, Melbourne, Victoria, 3122, Australia.

2National Computational Infrastructure, Australian National University, Canberra, ACT 0200, Australia

E-mail addresses: *fwang@swin.edu.au (F. Wang)

Supplementary Materials

Table S1: Geometric properties of Fc^+ (both conformers) using different models*

Parameter	UB3LYP/ m6-31G(d)[a]	B3LYP/ 6-31+G(d)[59]	B3LYP/ LANL2TZF[b]	UB3LYP/ DZVP[42]	B97-D/ 6-31+G(d)[59]	UHF/ m6-31G(d)[a]	UMP2/ m6-31G(d)[a]	MP2/ 6-31+G(d)[59]	Expt[60]
					**Eclipsed (D_{5h})**				
Fe-Cp (Å)	1.69 (0.02)	1.70 (0.02)	1.70 (0.02)	1.693(0.02)	1.71 (0.08)	1.80 (-0.05)	1.55 (0.07)	1.54	1.68 (0.02)
Fe-C (Å)	2.09 (0.02)	2.09 (0.02)	2.09 (0.02)	2.086(0.02)	2.10 (0.06)	2.17 (-0.04)	1.98 (0.06)	1.97	2.069
C-C (Å)	1.43 (0.0)	1.43 (0.0)	1.43 (0.0)	1.433(0.0)	1.44 (0.0)	1.41	1.44 (0.00)	1.44	
C-H (Å)	1.08 (0.0)	1.08 (0.0)	1.08 (0.0)	1.083(0.0)	1.09 (0.0)	1.07	1.08 (0.00)	1.08	
∡ Cp-H (°)	1.23	1.60			1.80	0.29	1.20	1.60	
$\langle R^2 \rangle$(a.u.)	1349.29					1438.74	1243.46		
					**Staggered (D_{5d})**				
Fe-Cp (Å)	1.70 (0.02)	1.70 (0.02)			1.71 (0.08)	1.80 (-0.05)	1.56 (0.06)		1.701 (0.04)
									1.71 (0.06)[d]
Fe-C (Å)	2.09 (0.02)	2.09 (0.02)			2.10 (0.06)	2.17 (-0.04)	1.98 (0.05)		2.046
									2.10[d]
C-C (Å)	1.43 (0.0)	1.43 (0.0)			1.43 (-0.01)	1.41	1.44 (0.00)	1.44	1.42[d]
C-H (Å)	1.08 (0.0)	1.08 (0.0)			1.09 (0.0)	1.07	1.08 (-0.01)	1.09	0.95[d]
∡ Cp-H (°)	1.48	1.80			2.0	0.36	1.72	1.90	
$\langle R^2 \rangle$(a.u.)	1350.30					1438.59	1246.91		
ΔE[c]	0.36	0.54				0.03	1.03		

* The variations (**$\Delta = Fc^+ - Fc$**) with respect to the corresponding Fc conformer using the same model are given in parenthesis.

[a] This work (m6-31G(d) for Fe, 6-31G(d) for C and H)

[b] Basic Set : LANL2TZF for Fe, 6-31G(d) for C and H (Ref [20])

[c] ΔE ($kcal\cdot mol^{-1}$) = $E_{tot}(D_{5d})$ - E_{tot} (D_{5h}).

[d] X-ray crystallography study (Ref [61])

Table S2: Benchmarking calculations for first IP (eV) of ferrocene using B3LYP, CCSD, and CCSD(T) and various basis sets[a,b].

Model	IP	Δ%
B3LYP/m6-31G(d)	6.902	0.61
B3LYP/m6-31G(d,p)	6.912	0.76
B3LYP/m6-31++G(d,p)	7.098	3.47
B3LYP/m6-31++G(2d,2p)	7.068	3.03
B3LYP/m6-31++G(3d,3p)	7.094	3.41
B3LYP/m6-31++G(3df,3pd)	7.099	3.48
CCSD/m6-31G(d)	6.854	-0.09
CCSD(T)/m6-31G(d)	6.763	-1.41
CCSD/m6-31G(d) – FULL	6.931	1.03
CCSD(T)/m6-31G(d) - FULL	6.844	-0.23
CCSD/m6-31G(d,p)	6.938	1.14
CCSD(T)/m6-31G(d,p)	6.777	-1.21
CCSD/m6-31++G(d,p)	7.121	3.80
CCSD(T)/m6-31++G(d,p)	6.983	1.79
CCSD/m6-31++G(2d,2p)	7.162	4.40
CCSD(T)/m6-31++G(2d,2p)	7.039	2.61
CCSD/m6-31++G(3d,3p)	7.210	5.10
CCSD(T)/m6-31++G(3d,3p)	7.107	3.60
CCSD/m6-31++G(3df,3pd)	7.252	5.71
CCSD(T)/m6-31++G(3df,3pd)	7.155	4.30
CCSD(T)/cc-pVDZ	6.981	1.76
CCSD(T)/m(aug-cc-pVDZ)	7.099	3.48
CCSD(T)/aug-cc-pVDZ	7.015	2.26
cc-pVTZ	7.024	2.39
m(aug-cc-pVTZ)	7.083	3.25
aug-cc-pVTZ	7.031	2.49

cc-pVQZ	7.024	2.39
aug-cc-pVQZ	7.029	2.46
CBS_{234}(cc-pVQZ)	7.019	2.32
CBS_{234}(aug-cc-pVQZ)	7.026	2.42
cc-pV5Z	7.026	2.42
aug-cc-pV5Z	7.027	2.43
CBS_{345}(cc-pV5Z)	7.028	2.45
CBS_{345}(aug-cc-pV5Z)	7.025	2.41
CCSD + m(aug-cc-pVDZ)	7.206	5.04
CCSD(t) + m(aug-cc-pVDZ)	7.173	4.56
CCSD + aug-cc-pVDZ	7.167	4.48
CCSD(t) + aug-cc-pVDZ	7.127	3.89
CCSD + m(aug-cc-pVTZ)	7.264	5.89
CCSD(t) + m(aug-cc-pVTZ)	7.251	5.70
CCSD + aug-cc-pVTZ		
CCSD(t) + aug-cc-pVTZ		
CCSD/pc-1	7.145	4.15
CCSD(T)/pc-1	7.004	2.10

[a]Based on B3LYP/m6-31G optimized geometries of eclipsed ferrocene (1 E_h = 27.2107 eV).
[b]Experimental measurement of the first IP of Fc is in the range of 6.72 eV-6.99 eV, depending on the experimental technique and conditions.

Table S3: Summarized information of the basis sets in the benchmarking calculations.

Basis	Basis Functions	Primitive Gaussians	Cartesian basis functions
m6-31G(d)	194	424	209
m6-31G(d,p)	224	454	239
m6-31++G(d,p)	274	504	289
m6-31++G(2d,2p)	354	594	379
m6-31++G(3d,3p)	434	684	469
m6-31++G(3df,3pd)	554	844	629
cc-pVDZ	233	694	249
m(aug-cc-pVDZ)	354	644	379
aug-cc-pVDZ	379	854	409
cc-pVTZ	508	1041	584
m(aug-cc-pVTZ)	724	1094	839
aug-cc-pVTZ	783	1376	919
cc-pVQZ	954	1779	1190
aug-cc-pVQZ	1400	2385	1796
cc-pV5Z	1613	2895	2184
aug-cc-pV5Z	2272	3889	3178

Figure S1: Summarized comparison about number of basic functions in the benchmarking calculations.

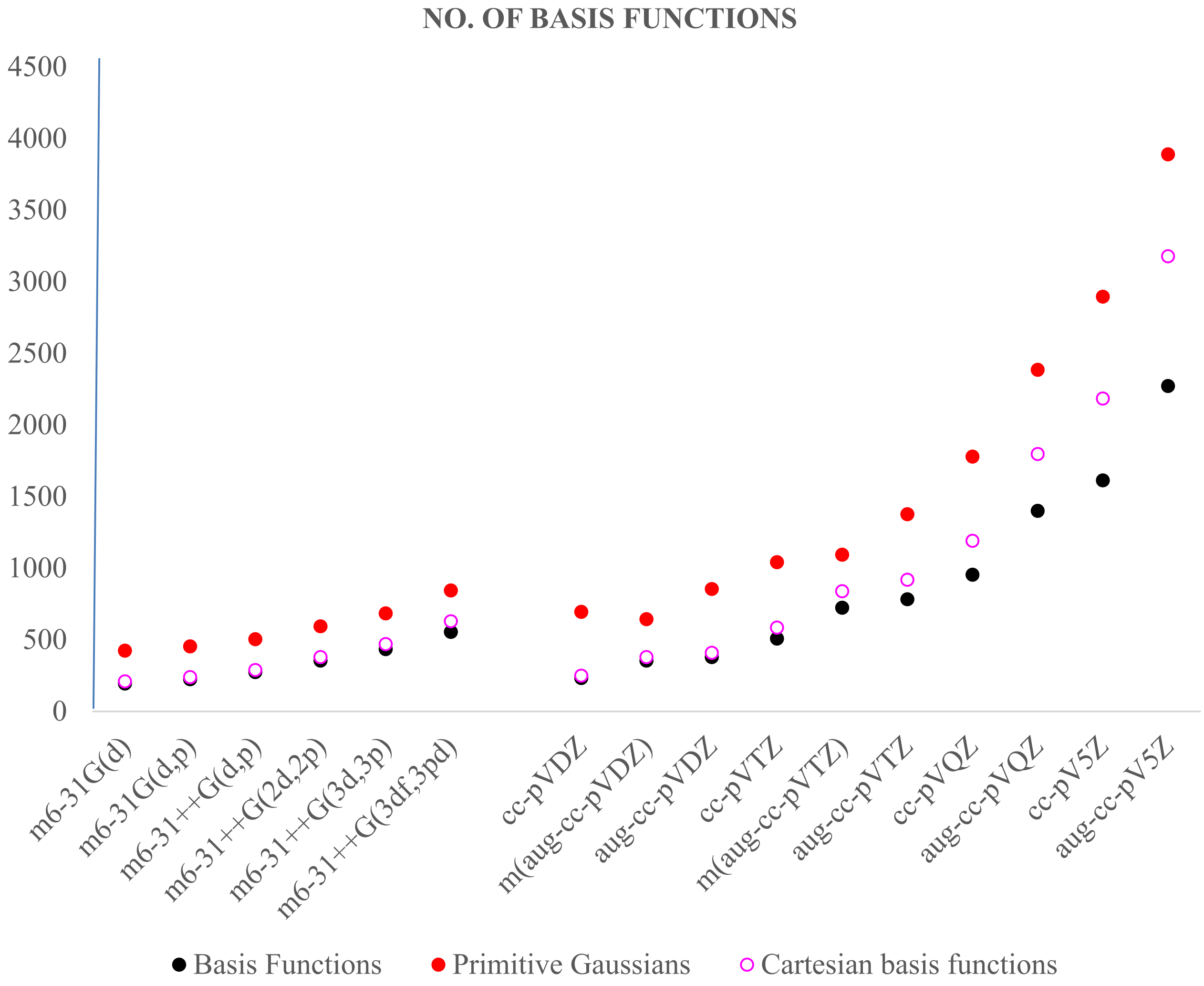

Figure S2 Comparison of the gold standard CCSD and CCSD(T) level of theory with various basis sets for the first AIP of Fc. The results (6.85 eV) with gold colors exhibit the best agreement with the recognised measurement.

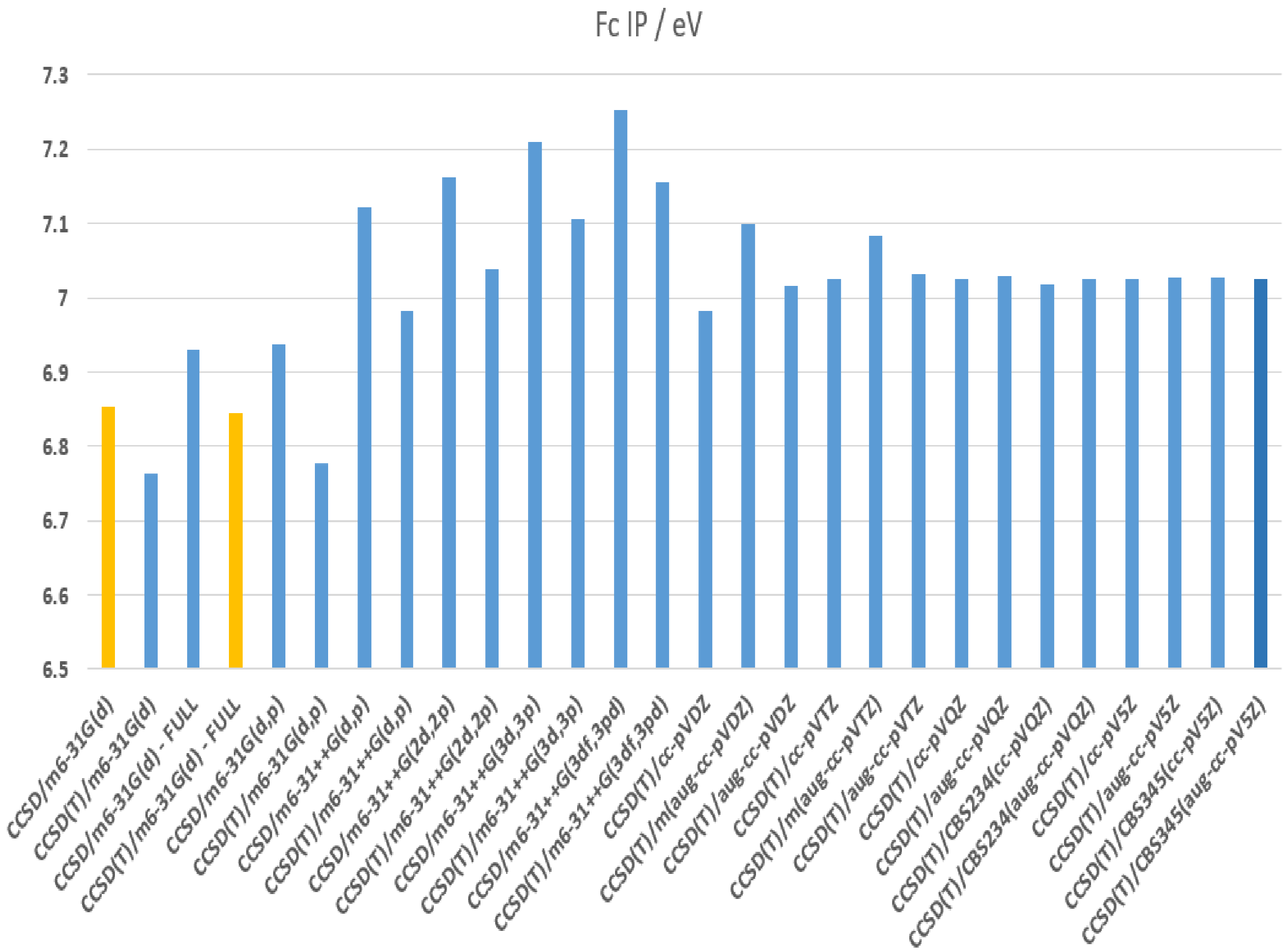

Supplement: Supplementary file 1 [file molecules-30-03541-s001.zip › molecules-3814472-supplementary.pdf]
